# Knowledge and practice of obstetric care providers on prevention of obstetric fistula 2023: an institution-based cross-sectional study

**DOI:** 10.3389/fgwh.2023.1234013

**Published:** 2023-11-30

**Authors:** Solomon Seyife Alemu, Mahlet Tesfaye Agago, Eshetu Yisihak Ukumo, Tesfahun Simon Hadero

**Affiliations:** ^1^ Department of Midwifery, Shashemenne Campus College of Health Science, Madda Walabu University, Shashemenne, Ethiopia; ^2^ Department of Midwifery, College of Health Science, Mattu University, Mattu, Ethiopia; ^3^ Department of Midwifery, College of Medicine and Health Science, Arba Minch University, Arbaminch, Ethiopia

**Keywords:** knowledge, practice, obstetric fistula, obstetric caregivers, Gamo zone

## Abstract

**Background:**

Obstetric fistula is a preventable devastating condition that is mostly caused by obstructed labour. About 22% of obstructed labor is complicated by obstetric fistula. Skilled birth attendants during delivery are essential for the prevention of obstetric fistula. However, little is known about the status of the knowledge and practice of obstetric fistula prevention in the Gamo zone.

**Objective:**

We aimed to assess the knowledge, practice, and associated factors of obstetric caregivers on the prevention of obstetric fistula in public health facilities of the Gamo zone in southwest Ethiopia 2023.

**Method:**

A cross-sectional study was employed among 372 obstetric caregivers in selected public health facilities of the Gamo zone in southwest Ethiopia from 1 December 2022 to 30 January 2023. Study participants were selected by a simple random sampling technique, and data were collected by using a pre-tested and self-administered questionnaire. The collected data were coded and entered into Epi-Data version 4.6 computer software and exported to SPSS version 27 for analysis purposes. Bivariable and Multivariable Logistic analyses were applied. The level of significance was declared at a *P*-value ≤0.05 and a 95% confidence interval.

**Results:**

About 57% [95% CI (53.00–62.00)] of participants had good knowledge, and about 55.4% [95% CI (50.00–60.00)] of obstetric caregivers showed good practice for obstetric fistula prevention. The factors significantly associated with knowledge were service year [AOR = 2.50, 95% CI = (1.12–6.73)], types of a health facility [AOR = 1.99, 95% CI = (1.01–3.92)], age [AOR = 2.38, 95% CI = (1.03–5.49)], and in-service training [AOR = 4.61, 95% CI = (2.35–9.05)]. In-service training [AOR = 14.86, 95% CI = (12.75–18.73)], service year [AOR = 3.58, 95% CI = (1.24–10.29)], and knowledge [AOR: 13.24, 95% CI = (6.18–14.34)] were factors which were significantly associated with the practice of obstetric caregivers towards obstetric fistula prevention.

**Conclusion:**

The knowledge and practice of obstetric caregivers on the prevention of obstetric fistula was low in public health facilities of the Gamo zone. In this study, practicing at a hospital was a factor significantly associated with the knowledge of obstetric caregivers. Having in-service training, advanced service year, and age were factors significantly associated with the knowledge and practice of obstetric caregivers. Regular in-service training of health professionals can enhance their knowledge and practice of obstetric fistula prevention.

## 1. Background

Obstetric fistula (OF) is an abnormal communication between the vagina and the bladder or between the vagina and the rectum or both that leads to uncontrollable leakage of urine and feces through the openings (1). There are many classifications for OF, but the most common are vesicovaginal fistula (VVF) and rectovaginal fistula (RVF). Since there is improved obstetric care, OF is not prevalent in high-income countries, but it remains a prevalent cause of maternal morbidity and mortality in low-income countries (2–4).

According to World Health Organization (WHO) estimates, over 2 million females are living with untreated OF worldwide, with 50,000–100,000 new cases reported each year. Of these, about 1 million women live in northern Nigeria, over 70,000 women live in Bangladesh, and around 26,000–40,000 women live in our country, Ethiopia (1, 3, 5). But these figures are likely to be underestimated because they include only those mothers who seek care (1, 3, 6).

Worldwide, about 2–3 million young women are living with obstetric fistula, and 50,000–10,000 new cases per year were reported. In sub-Saharan Africa, about 30,000–13,000 new cases are registered and reported (1, 7). According to the EDHS 2016 report, in Ethiopia, only 4 in 10 women aged 15–49 (39%) have heard of obstetric fistula (5). In Ethiopia, many thousands of women are still living with fistula (1, 8–10). A population-based survey in Ethiopia using a questionnaire as a proxy to estimate obstetric fistula prevalence in 2005 showed that the prevalence of Obstetric Fistula in the South Nation and Nationalities People Region (SNNPR) was 1.5% (7).

Obstetric fistula is a serious, long-term, life-altering obstetric complication in developing countries and is considered a marker of poor basic health care service. The consequence of obstetric fistula is not only the physical trauma and future birth complications; women face Psycho-social problems like being abandoned by families and friends and being stigmatized and discriminated against, which has led to depression, loneliness, and loss of self-esteem, self-worth, and identity. In addition, their fate is extreme poverty. It usually follows prolonged and neglected obstructed labor, which accounts for 8% of maternal deaths and 22% of obstructed labor complicated with obstetric fistula (11–14).

Even though obstetric fistula is preventable with simple and effective technology for monitoring the progress of labor that is readily available in health facilities, observational studies revealed that it is either not being used at all or incorrectly used by obstetric caregivers; they prefer to write words instead of using the partograph for decision making during labor and delivery (15, 16). In addition, healthcare providers keep laboring mothers at public health centers beyond the limited time for normal labor and refer them after it is too late with a full bladder, which contributes to obstetric fistula (17).

Ethiopia has made great strides in combating obstetric fistula, for example, expanding the primary health care system, expanding free maternity care at governmental health facilities, increasing transportation systems, and providing access to skilled birth attendants. However, the problem still affects the physical, social, and economic factors of women as well as the country. In 2014, the government initiated a 5-year campaign against obstetric fistula. Non-Government Organizations (NGOs) support the government's efforts to provide thousands of Ethiopian women with the obstetric healthcare they need. Chief among these organizations is the Hamlin Fistula Hospital, the global leader in fistula care (18, 19). The United nation fund for population agency (UNFPA) collaborates with both governmental and non-governmental stakeholders and launched a global campaign in 2003 to end obstetric fistula by 2020 with the aim of treatment and social rehabilitation for those women with obstetric fistula. Even though strategies exist, they need further development of prevention strategies rather than treatments (17, 20). Assessing the knowledge and practice of obstetric care providers regarding obstetric fistula prevention provides valuable insights regarding obstetric fistula prevention in the region. There were a few pockets of studies conducted regarding the prevention of obstetric fistula in Ethiopia. In contrast to the preceding studies, the study participants in these studies were enrolled from both urban and rural health facilities. So this study aimed to assess the knowledge, practice, and associated factors of obstetric care providers on obstetric fistula prevention.

## 2. Methods and materials

### 2.1. Study area and period

This study was conducted in selected public health facilities of the Gamo Zone, Southwest Ethiopia. Arba Minch is located 505 kms from the South of Addis Ababa, the capital city of Ethiopia, and about 275 km from Hawassa, the capital of the SNNP region. The Gamo people are an Ethiopian ethnic group located in the Gamo Highlands of Southwest Ethiopia. They are found in more than 40 communities, including Chencha, Bonke, Kucha, Garbansa, Zargula, Kamba, Dorze, Birbir, Ochello, Boroda, Ganta, Gacho Baba, Eligo, Shella, Kolle, Dita, Kogo, and Daramalo. The population of the Zone was 1,544,753 people of which 51% were women in 2020 (21). The Gamo Zone has 15 woredas (districts) and two town administrations, each being directly accountable to the zone. The zone has six hospitals and 57 health centers, and the zone also had 88% and 77% of ANC coverage and family planning coverage, respectively, in 2013 (21). According to the zonal health office report, the total number of health professionals serving health facilities in the Gamo zone were 606. The study was conducted from 1 December 2022 to 30 January 2023.

### 2.2. Study design

An institution-based cross-sectional study was employed.

### 2.3. Population

#### 2.3.1. Source population

Our source population was all obstetric caregivers who were employed and currently working in public health facilities in the Gamo Zone.

#### 2.3.2. Study population

Our study population consisted of selected obstetric caregivers who were working in selected public health facilities of the Gamo Zone.

### 2.4. Eligibility criteria

Individuals who have worked for at least 6 months in public health facilities located in the Gamo Zone were included in this study whereas individuals who were on maternity leave were excluded.

### 2.5. Sample size determination and sampling techniques

#### 2.5.1. Sample size determination

The sample size was determined using a single population proportion formula by taking the proportions of 67% and 66.2% for knowledge and practice, respectively, from a study conducted in Addis Ababa, Ethiopia, with a confidence level of 95% and a margin of error of 5%; the sample size was 340 for knowledge and 345 for practice for obstetric caregivers on prevention of obstetric fistula (22).


$$\begin{array}{c} n=\frac{Z^{2}p[1-P]}{d^{2}}=340\\ n=\frac{Z^{2}p[1-P]}{d^{2}}=345 \end{array}$$

By adding a 10% non-response rate, the final sample size (*n*) was **380**.

#### 2.5.2. Sampling technique and procedure

**Figure 1:**
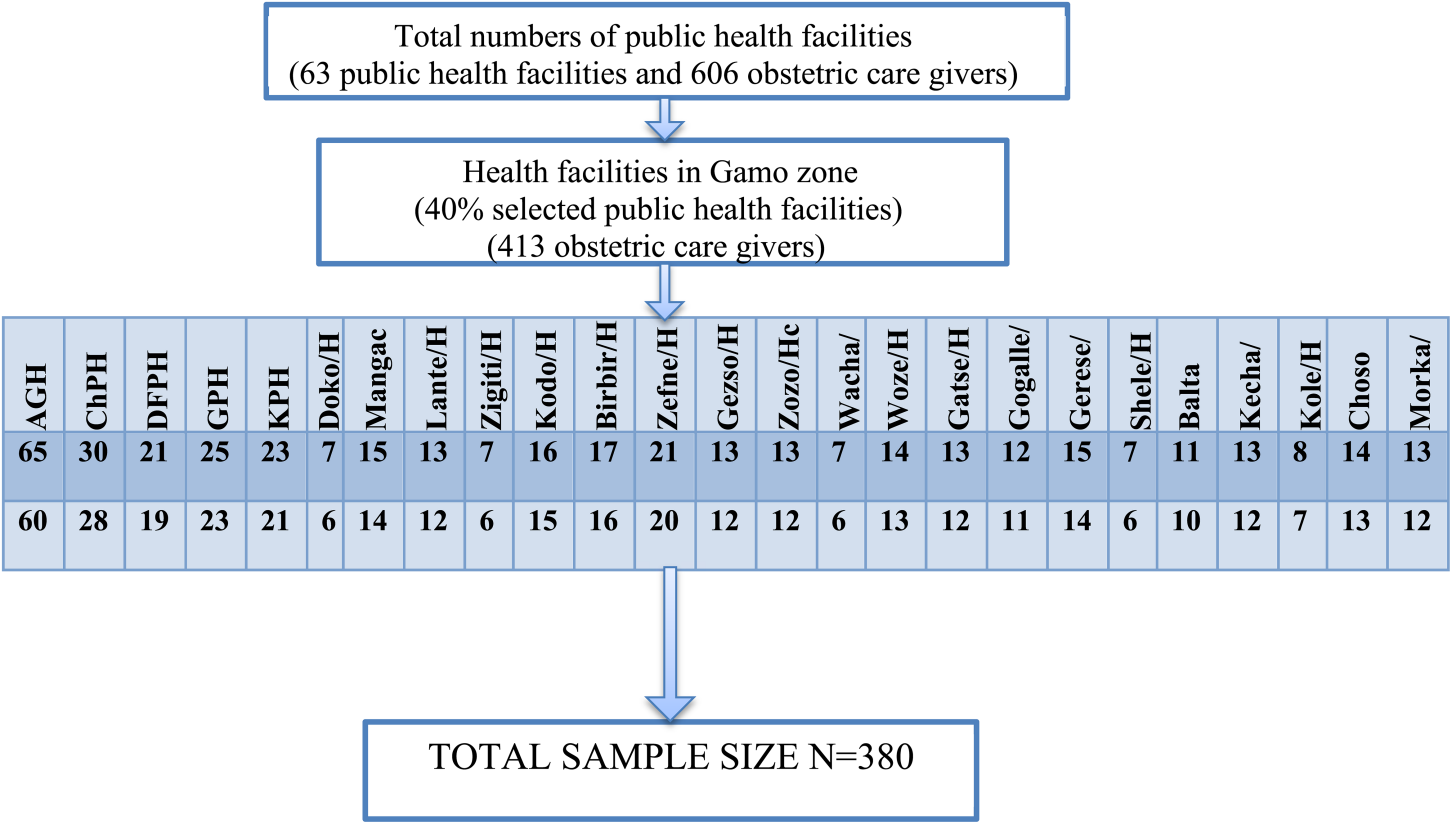
A schematic illustration of sample allocation in selected public health facilities of the Gamo zone, southwest Ethiopia, 2023.

The study was conducted among obstetric caregivers found in Gamo zone public health facilities. According to the zonal health bureau information, the Gamo zone has a total of 63 public health facilities with 606 health care providers working in the obstetrics unit. By taking WHO's recommendation on sampling techniques, 40% of health facilities were included in the study. So, from the total of 63 public health facilities found in the zone, 25 (40%) health facilities were selected by using the lottery method. The total number of healthcare providers that are working at an obstetric unit in selected public health facilities that was obtained from the zonal health bureau was 413. Before the selection of study participants, proportionate allocations to each health facility were carried out. Subsequently, the list of obstetric caregivers was obtained from the head of the maternity department of each health facility. Lastly, the simple random sampling lottery method was used at each health facility to select a proportionate number of obstetric caregivers (Figure 1).

### 2.6. Variables of the study

#### 2.6.1. Dependent variable

Knowledge of the prevention of obstetric fistula and practice on prevention of obstetric fistula.

#### 2.6.2. Independent variables

Factors associated with knowledge and practice on prevention of obstetric fistula include the following: socio-demographic characteristics, such as age, sex, marital status, and religion, and occupational characteristics, such as professional qualification, service year, working part-time, and the level of the profession. In addition, training- and health-facility-related factors include pre-service training, in-service training on prevention of Obstetric Fistula [Partograph, family planning, and Basic Emergency Obstetric and Newborn Care (BEmONc)], and types of health facility.

### 2.7. Data collection procedures and tools

Data were collected using a structured self-administered questionnaire to assess knowledge and an observational checklist for practice assessment. The data collection tools were adopted from Prevention and Management of Obstetric Fistula: A Curriculum for Nurses and Midwives 2012, East, Central, and Southern African Health Community (ECSA-HC) and Engender Health/Fistula Care (23).

The questionnaire has three parts. The first part contained socio-demographic information including professional qualifications and years of service. The second part of the questionnaire contained variables to assess the knowledge and predisposing factors to obstetric fistula. The final part of the checklist concerned the practice of the participants. In data collection and supervision, four clinical midwifery masters and two public health masters participated, respectively. Re-visit and call-back arrangements were made in situations where there was a high workload during data collection.

### 2.8. Data quality assurance

To assure the quality of the data, a pre-test was carried out on a sample of 38 obstetric caregivers (10% of the sample size) in the Wolaita Sodo Otona referral hospital. The internal consistency of the tool was assessed by the reliability test (Cronbach's alpha). The values of Cronbach's alpha were 0.906 and 0.919 for knowledge and practice questions, respectively.

The training was given for 2 days for data collectors and supervisors on the objective and issues of confidentiality and privacy. Supervisors oversaw the data collection process daily. At the end of every data collection day, each questionnaire was examined for completeness and consistency by the supervisors and the principal investigator, and pertinent feedback was given to the data collectors and supervisors.

### 2.9. Data processing and analysis

The collected data were coded and entered into Epi-Data version 4.6 software and exported to SPSS statistical software version 27 for data cleaning and further analysis. Errors related to the inconsistency of data were checked and corrected during data cleaning. The test of Normality was checked to select the appropriate statistical summary measure. Descriptive statistical analyses such as simple frequencies, percentages, mean, and standard deviation were used to describe the characteristics of participants. For further analysis, the association between an outcome variable and each independent variable was seen separately in a binary logistic regression model. Variables with a *p*-value of less than 0.25 in Bivariable analyses were retrieved and entered for multivariable logistic regression analyses. Multi-colinearity was checked to see the linear correlation among the independent variables by correlation coefficient and variance inflation factors. The degree of association between an outcome variable and independent variables was determined using an adjusted odds ratio along with 95% CI and *p*-value. The level of statistical significance was declared at a *p*-value less than 0.05. The fitness of the model was tested by Hosmer–Lemeshow's goodness-of-fit test model; the values were 0.89 and 0.95 for knowledge and practice, respectively. Finally, the result was presented by text, table, and graph.

### 2.10. Operational definitions

**Good Knowledge**: there were 11 questions and their responses to assess knowledge of obstetrical fistula; the average number of knowledge questions that respondents answered correctly was calculated, and those who scored the mean or above were considered knowledgeable (22).

**Poor knowledge:** there were 11 questions and their responses to assess knowledge of obstetrical fistula; the average number of knowledge questions that respondents answered correctly were calculated, and those who scored the below mean were considered as having poor knowledge (22).

**Good Practice**: the average number of practice items that participants practiced correctly were calculated, and those who scored 100% were considered to show good practice.

**Poor Practice:** the average number of practice items that the participants practiced correctly were calculated, and those who scored below 100% were considered to show poor practice.

**In-service training:** a regular process to refresh and update the skills, competence, and knowledge of health care providers in key areas like BEmoNC and partograph at least once, which is basic obstetrics fistula prevention (22).

**Pre-service training:** this indicates the site where they trained as health care providers in terms of private or governmental institutions (22).

## 3. Results

### 3.1. Socio-demographic characteristics of the study participants

In this study, 372 obstetric caregivers volunteered to give information making a response rate of 97.9%. The mean age of the respondents was 29 years [SD ± 3]. Regarding marital status, 166 (44.6%) of the study participants were married, and 148 (39.8%) of respondents were single. Among the total respondents, 59.9% were protestant religious followers (Table 1).

**Table 1:** Socio-demographic characteristics of the obstetric care providers in selected public health facilities of the Gamo zone, southwest Ethiopia 2023 (*n *=* *372).

Socio-demographic characteristics	Frequency	Percent (%)
Age in years categories		
<25	52	14
26–34	266	71.5
>35	54	14.5
Sex		
Male	113	30.4
Female	259	69.6
Marital status		
Single	148	39.8
Married	166	44.6
Widowed	58	15.6
Religion		
Protestant	223	59.9
Orthodox	104	28
Muslim	45	12.1

### 3.2. Occupational-related characteristics

In this study, about two-thirds of the 273 (73.4%) obstetric caregivers were midwives. Regarding the level of profession (cadre), 197 (53%) respondents had a Bachelor of Science degree. Among all obstetric caregivers, 111 (29.8%) were working in a private clinic. Concerning the year of service, 125 (33.6%) of respondents had fewer than 3 years of experience (Table 2).

**Table 2:** Occupational-related characteristics of the obstetric caregivers in selected public health facilities of the Gamo zone, southwest Ethiopia 2023 (*n *=* *372).

Occupational-related characteristics	Frequency	Percent (%)
Professional qualification		
General practitioner	50	13.4
Midwifery	273	73.4
Nurse	49	13.2
Level of profession		
Diploma	175	47
Degree	197	53
Service year		
<3	125	33.6
3–6	104	28
6–10	92	24.7
>11	51	13.7
Working part-time		
Yes	111	29.8
No	261	70.2

### 3.3. Training and health facility-related characteristics

In terms of the training-related characteristics of respondents, almost three-fourths were trained as a Health Care Provider (HCP) in government universities and colleges. A total of 176 respondents experienced in-service training regarding BEmoNC and partograph. Among all obstetric caregivers, 151 (40.6%) respondents were working in public hospitals (Table 3).

**Table 3:** Training and health facility-related characteristics of the obstetric caregivers in selected public health facilities of the Gamo zone, southwest Ethiopia 2023 (*n *=* *372).

Training and health facility-related factors	Frequency	Percent
Pre-service training		
Government	265	71.2
Private	107	28.8
In-service training		
Yes	176	47.3
No	196	52.7
Health facility		
Hospital	151	40.6
Health center	221	59.4

### 3.4. Knowledge of obstetric caregivers on prevention of obstetric fistula

In terms of the knowledge about obstetric fistula prevention, 212 (57%) participants had good knowledge about obstetric fistula prevention. Several of the respondents, 160 (43%), knew the use of partograph during labor. About 269 (72.3) respondents knew the duration of normal labor (Table 4).

**Table 4:** Knowledge of obstetric caregivers towards prevention of obstetric fistula in (*n *=* *372) selected public health facilities of the Gamo zone, southwest Ethiopia 2023.

Items to assess knowledge	Responses	Frequency	Percent
Duration of normal labor	More than 24 h	269	72.3
	Less than 24 h	103	27.7
Use of partograph prevents OF	Yes	160	43
	No	212	57
Early identification of obstructed labor prevents OF	Yes	159	42.7
	No	213	57.3
Younger age is a factor	Yes	258	69.4
	No	114	30.6
Early marriage is a factor	Yes	260	69.9
	No	112	30.1
Childhood malnutrition is a factor	Yes	266	71.3
	No	106	28.5
Rehydration is useful to prevent OF	Yes	266	28.5
	No	106	71.5
The use of family planning prevents the occurrence of obstetric fistula	Yes	256	68.8
	No	116	31.2
Access to maternity services prevents OF	Yes	145	61
	No	227	39
Obstetric caregivers have a role to prevent	Yes	141	37.9
	No	231	32.1
Adequate coverage of topic during pre-service training	Yes	148	39.8
	No	224	60.2

### 3.5. Practice of obstetric caregivers on prevention of obstetric fistula

In terms of the practice of obstetric fistula prevention, 206 (55.4%) participants showed good practice of obstetric fistula prevention.

### 3.6. Factors associated with knowledge of obstetric caregivers

The model fitness test was checked using Hosmer–Lemeshow goodness-of-fit with a *P*-value of 0.89. In this study, seven variables were a candidate for multivariable analysis. Four variables, age, types of health facility, in-service training, and service year, were significantly associated with obstetric caregivers having knowledge of obstetric fistula prevention.

The odds of having good knowledge among participants who are working at hospitals were 2 times [AOR: 1.99; 95% CI 1.01–3.92)] higher compared to those who are working in public health centers. The other significant variable was in-service training regarding the partograph and BEmONc. The odds of having good knowledge among respondents who have in-service training were 4.6 times [AOR: 4.61; 95% CI 2.35–9.05] higher than for those who have no in-service training. Similarly, the odds of having good knowledge among participants who have >11 years of experience were 2.5 times [AOR: 2.50, 95% CI 1.12–6.73] higher than for those who have <3 years of experience (Table 5).

**Table 5:** Bivariable and multivariable logistic regression analysis of the knowledge of obstetric caregivers on prevention of obstetric fistula in public health facilities of the Gamo zone, southwest Ethiopia 2023 (*n *=* *372).

Variables	Knowledge		Crude odds ratio (95% CI)	Adjusted odds ratio 95% CI)	*P*-Value
	Good	Poor			
Age					
<25	15 (7.1%)	37 (23.1%)	1	1	
26–34	167 (78.8%)	99 (61.9%)	4.16 (2.17–7.96)	**2.38 (1.03–5.49)**	**0.04** *
>34	30 (14.2%)	24 (15%)	3.08 (1.37–6.89)	1.91 (1.32–4.99)	0.87
Marital status					
Single	110 (51.9%)	38 (23.8%)	1	1	
Married	78 (36.8%)	88 (55%)	0.30 (0.19–0.49)	0.54 (0.27–0.82)	0.08
Divorced	24 (11.3%)	34 (21.3%)	0.24 (0.15–0.46)	0.57 (0.23–0.98)	0.2
Health facility					
Hospital	108 (50.9%)	43 (26.9%)	2.83 (1.82–4.39)	**1.99 (1.01–3.92)**	**0.047** *
Health center	104 (49.1%)	117 (73.1%)	1	1	
Professional qualification					
General practitioner	39 (18.4%)	11 (6.9%)	3.69 (1.54–8.83)	1.39 (1.11–8.84)	0.14
Midwifery	149 (70.3%)	124 (77.5%)	1.25 (1.08–2.30)	1.05 (1.03–6.90)	0.1
Nurse	24 (11.3%)	25 (15.6%)	1	1	0.1
Working part-time					
Yes	52 (24.5%)	59 (36.9%)	0.55 (0.35–0.81)	0.86 (0.44–0.96)	0.65
No	160 (75.5%)	101 (63.1%)	1	1	
In-service training					
Yes	150 (70.8%)	26 (16.3%)	12.46 (7.00–20.00)	**4.61 (2.35–9.05)**	**<0.001** *
No	62 (29.2%)	134 (83.8%)	1	1	
Service year					
<3	60 (28.3%)	65 (40.6%)	1	1	
3–6	68 (32.1%)	36 (22.1%)	2.04 (1.19–3.49)	1.54 (1.24–8.94)	0.1
6–10	45 (21.2%)	47 (29.4%)	1.03 (1.01–5.99)	1.02 (1.01–3.77)	0.8
>11	39 (18.4%)	12 (7.5%)	3.52 (1.68–7.35)	**2.50 (1.12–6.73)**	**<0.001** *

* Bolded means variables with *p*-values <0.05.

### 3.7. Factors associated with the practice of obstetric caregivers

The model fitness test was checked using Hosmer–Lemeshow goodness-of-fit with a *P*-value of 0.95. In this study, 9 variables were a candidate for multivariable analysis. In-service training, service year, age, and knowledge were significantly associated with obstetric caregivers having practice with obstetric fistula prevention. The odds of showing good practice among participants who have in-service training were 15 times [AOR: 14.86; 95% CI (12.75–18.73)] higher than for those who have no in-service training, and the odds of showing good practice among participants who have good knowledge were 13 times [AOR = 13.24; 95% CI (6.18–14.34)] higher than for those who have poor knowledge. Similarly, the odds of showing good practice among participants who have >11 years of experience were 3.6 times [AOR = 3.58; 95% CI (1.24–10.29)] higher than for those who have <3 years of experience (Table 6).

**Table 6:** Bivariable and multivariable logistic regression analysis of the practice of obstetric caregivers of prevention of obstetric fistula in public health facilities of the Gamo zone, southwest Ethiopia 2023 (*n *=* *372).

Variables	Knowledge		Crude odds ratio (95% CI)	Adjusted odds ratio 95% CI)	*P*-Value
	Good	Poor			
Age					
<25	5 (2.4%)	47 (28.3%)	1	1	
26–34	176 (85.4%)	90 (54.2%)	18.38 (7.06–27.83)	12.59 (9.09–18.71)	0.13
>34	25 (12%)	29 (17.5%)	8.10 (2.79–13.52)	**2.76 (2.00–10.6)**	**<0.001** *
Marital status					
Single	103 (50%)	45 (27.1%)	1	1	
Married	74 (36.8%)	92 (55.4%)	0.35 (0.22–0.56)	0.23 (0.18–0.87)	0.2
Divorced	29 (14.1%)	29 (17.3%)	0.44 (0.23–0.81)	0.34 (0.26–0.99)	0.8
Level of profession					
Diploma	50 (24.3%)	125 (75.3%)	1	1	
Degree	156 (75.7%)	41 (24.7%)	9.51 (5.91–15.30)	1.84 (1.06–4.13)	0.13
Health facility					
Hospital	112 (54.4%)	39 (23.5%)	3.88 (2.47–6.09)	1.87 (1.04–4.06)	0.1
Health center	94 (45.6%)	127 (76.5%)	1	1	
Professional qualification					
GP	39 (18.9%)	11 (6.6%)	4.00 (1.67–9.59)	2.80 (1.23–4.09)	0.9
Midwifery	144 (69.9%)	129 (77.7%)	1.26 (1.08–2.32)	1.08 (1.02–9.95)	0.6
Nurse	23 (11.2%)	26 (15.7%)	1	1	
Working part-time					
Yes	45 (21.8%)	66 (39.8%)	1	1	
No	161 (78.2%)	100 (60.2%)	0.42 (0.26–0.66)	0.24 (0.14–0.95)	0.8
In-service training					
Yes	159 (77.2%)	17 (10.2%)	29.65 (16.30–35.92)	**14.86 (12.75–18.73)**	**<0.001** *
No	47 (22.8%)	149 (89.8%)	1	1	
Service year					
<3	39 (18.9%)	86 (51.8%)	1	1	
3–6	73 (35.4%)	31 (18.7%)	5.19 (2.45–9.14)	3.02 (2.20–13.84)	0.46
6–10	56 (27.2%)	36 (21.7%)	3.43 (1.95–6.03)	2.42 (2.00–14.33)	0.49
>11	38 (18.4%)	13 (7.8%)	6.44 (3.09–13.43)	**3.58 (1.24–10.29)**	**0.018** *
Knowledge					
Good	174 (84.5%)	38 (22.9%)	18.31 (10.86–23.89)	**13.24 (6.18–14.34)**	**<0.001** *
Poor	32 (15.5%)	128 (77.1%)	1	1	

* Bolded means variables with *p*-values <0.05.

## 4. Discussion

The overall knowledge level of obstetric care providers in this study was 57% [95% CI (53.00–62.00)]. This study shows higher knowledge levels than the studies conducted in Addis Ababa, Ethiopia (22), Eastern Nepal (24), Ibadan Nigeria (25), and Nigeria Calabar Teaching Hospital (26), which were 67.7%, 35%, 38.1%, and 13.5%, respectively. The discrepancy of this study from the Addis Ababa, Ethiopia, study might be due to the former study being conducted in a town where more experienced staff and advanced infrastructure existed. Furthermore, the differences from the Nepal and Nigeria studies might be attributed to differences in the study areas, which might be explained by differing strategies in and commitments to implementing the health policy at the various levels throughout the countries, and the infrastructure, health setup, and obstetric care providers for the prevention of obstetric fistula is varied.

The knowledge of obstetric caregivers in this study is lower than in the study conducted in Ghana, which was 86.7% (27). The discrepancy could be due to the difference in the study setting and study participants; the preceding studies were conducted among midwives only whereas this study was conducted among obstetric caregivers including other professions. Additionally, the previous study was conducted in an area with a fistula center, and awareness of obstetric fistula could therefore be higher in this population.

The overall practice of obstetric care providers in this study was 55.4% [95% CI (50.00−60.00)]. The value in this study is lower than that of the research conducted in Addis Ababa, Ethiopia, which was 66.2% (22). This might be due to the difference in the study setting and differences in methods by which practice was assessed. In the previous study, the practice was assessed by using self-administered questionnaires, whereas ours is through study.

Service year was one of the factors associated with knowledge of obstetric caregivers. Obstetric caregivers who have >11 years of experience were 2.5 times more likely to have good knowledge compared to those who have <3 years of experience This is supported by the study conducted in Ibadan, Nigeria (25). This might be due to experienced healthcare providers exposing themselves to different daily cases, updating themselves via training, which they may do more than their juniors, as well as acquiring knowledge through informal (from their colleagues and seniors) and formal learning (updating their rank of education) throughout their years of service.

Participants who are working at hospitals were two times more likely to have good knowledge compared to those who are working in health centers. This is supported by the study conducted in Ibadan, Nigeria (25). The possible reason for this could be that obstetric caregivers in hospitals have more training than in health centers, and they communicate with their seniors on the different cases they have faced. Furthermore, hospital staff have experience because they are challenged daily by the referral cases.

Another significant factor in this study was in-service training regarding the partograph and BEmONc. Respondents who have in-service training were 4.6 times more likely to have good knowledge compared to their counterparts. This is supported by the study conducted in Addis Ababa, Ethiopia and a study conducted in Ibadan, Nigeria (22, 25). This might be because individuals who have on-job training had an opportunity to update their knowledge.

Knowledge about obstetric fistula prevention was significantly higher among obstetric care givers within the age group 26–34 years compared to those younger than 25 years. It might be because as the age of an individual increases, the probability of acquiring comprehensive knowledge of obstetric fistula prevention should also increase. This could be related to experience or in-service training on obstetric fistula prevention having a positive relationship with knowledge of obstetric fistula prevention.

This study also revealed that participants who have in-service training were 15 times more likely to display good practice compared to those who have no in-service training. This is supported by the study conducted in Addis Ababa, Ethiopia (22). This finding points to the need for obstetric caregivers to receive periodic on-job refresher training on obstetric fistula prevention.

The odds of good practice among participants who have good knowledge were 13 times higher compared to their counterparts. This is supported by the study conducted in Addis Ababa, Ethiopia (22). The observed finding is expected because the higher the level of a person's awareness the better chance they have of implementing their knowledge.

The odds of good practice among participants who have >11 years of experience were 3.6 times higher compared to those who have <3 years of experience. This is supported by the study conducted in Addis Ababa, Ethiopia (22). This might be due to the fact that the experienced health care providers can improve their status of practice through informal and formal learning, and the longer they stay in their profession the more chances they have of getting in-service training, which contributes to developing their skills.

The odds of good practice among obstetric care givers within the age group of those >34 years old were 2.76 times higher compared to those who are in the age group of <25. This could be related to the fact that, as the age of an individual increases, they might become experienced and become the only one to be consulted by their junior obstetric caregivers, and thus it might enable them to update their knowledge and skills.

### 4.1. Strength and limitations

Unlike the previous study conducted in Ethiopia, where the method of data collection to assess practice to prevent obstetric fistula was a self-filled checklist, this study used multiple observations to assess the practice and helps to minimize observational bias. However, there was scarcity of similar studies despite the efforts made, and the study was exposed to the Hawthorne effect, which occurs when a participant's behavior changes as a result of being observed, varying from their actual practices. To overcome this challenge as much as possible, experienced data collectors and supervisors were used.

## 5. Conclusions and recommendations

The knowledge and practice of obstetric caregivers on the prevention of obstetric fistula is low in the public health facilities of the Gamo Zone. In this study, service year, practicing at a hospital, advanced age, and having in-service training were factors significantly associated with knowledge of obstetric caregivers. On the other hand, having in-service training, advanced service year, advanced age, and having good knowledge were factors significantly associated with the practice of health care providers.

Obstetric caregivers with little professional experience would receive more attention through the provision of in-service training related to obstetric fistula prevention to increase their level of knowledge and practice towards obstetric fistula during their year of practice and they would also emphasize the proficient composition of staff among those who are working in health centers. Non-Government Organizations and professional associations would support and contribute to updating the knowledge and practice of obstetric caregivers through the provision of in-service training on the topics that contribute to the prevention of obstetric fistula. In addition, the Gamo zonal health office and other concerned bodies bring attention to the need to enhance prevention of obstetric fistula by providing a chance for training, resource allocation, and collaboration with different stakeholders. Furthermore, we would like to recommend that upcoming researchers should incorporate intra-operation and post-operation obstetric fistula prevention studies for more generalizable information.

## Data Availability

The original contributions presented in the study are included in the article/Supplementary Material, further inquiries can be directed to the corresponding author.
